# Bulky DNA Adducts in Cord Blood, Maternal Fruit-and-Vegetable Consumption, and Birth Weight in a European Mother–Child Study (NewGeneris)

**DOI:** 10.1289/ehp.1206333

**Published:** 2013-07-23

**Authors:** Marie Pedersen, Bernadette Schoket, Roger W. Godschalk, John Wright, Hans von Stedingk, Margareta Törnqvist, Jordi Sunyer, Jeanette K. Nielsen, Domenico F. Merlo, Michelle A. Mendez, Helle M. Meltzer, Viktória Lukács, Anette Landström, Soterios A. Kyrtopoulos, Katalin Kovács, Lisbeth E. Knudsen, Margaretha Haugen, Laura J. Hardie, Kristine B. Gützkow, Sarah Fleming, Eleni Fthenou, Peter B. Farmer, Aina Espinosa, Leda Chatzi, Gunnar Brunborg, Nigel J. Brady, Maria Botsivali, Khelifa Arab, Lívia Anna, Jan Alexander, Silvia Agramunt, Jos C. Kleinjans, Dan Segerbäck, Manolis Kogevinas

**Affiliations:** 1 Centre for Research in Environmental Epidemiology (CREAL), Barcelona, Spain; 2 CIBER Epidemiología y Salud Pública (CIBERESP), Madrid, Spain; 3 INSERM (National Institute of Health and Medical Research), U823, Team of Environmental Epidemiology Applied to Reproduction and Respiratory Health, Institute Albert Bonniot, Grenoble, France; 4 Department of Molecular Environmental Epidemiology, National Institute of Environmental Health, Budapest, Hungary; 5 Department of Toxicology, Maastricht University, Maastricht, the Netherlands; 6 Bradford Institute for Health Research, Bradford Royal Infirmary, Bradford, United Kingdom; 7 Department of Materials and Environmental Chemistry, Environmental Chemistry Unit, Stockholm University, Stockholm, Sweden; 8 IMIM (Hospital del Mar Research Institute), Barcelona, Spain; 9 Universitat Pompeu Fabra, Departament de Ciències Experimentals i de la Salut, Barcelona, Spain; 10 Department of Public Health, University of Copenhagen, Copenhagen, Denmark; 11 Epidemiology, Biostatistics, and Clinical Trials, IRCCS AOU San Martino–IST (National Cancer Research Institute), Genova, Italy; 12 Department of Nutrition, University of North Carolina at Chapel Hill, Chapel Hill, North Carolina, USA; 13 Department of Exposure and Risk Assessment, Division of Environmental Medicine, Norwegian Institute of Public Health, Oslo, Norway; 14 Department of Biosciences and Nutrition, Karolinska Institutet, Novum, Huddinge, Sweden; 15 National Hellenic Research Foundation, Institute of Biology, Medicinal Chemistry and Biotechnology, Athens, Greece; 16 Leeds Institute of Genetics, Health and Therapeutics, University of Leeds, Leeds, United Kingdom; 17 Department of Chemicals and Radiation, Division of Environmental Medicine, Norwegian Institute of Public Health, Oslo, Norway; 18 Department of Social Medicine, Faculty of Medicine, University of Crete, Heraklion, Greece; 19 Department of Cancer Studies and Molecular Medicine, Biocentre, University of Leicester, United Kingdom; 20 Division of Epigenomics and Cancer Risk Factors, German Cancer Research Center (DKFZ), Heidelberg, Germany; 21 Office of the Director, Division of Environmental Medicine, Norwegian Institute of Public Health, Oslo, Norway; 22 Department of Toxicogenomics, Maastricht University, Maastricht, the Netherlands; 23 National School of Public Health, Athens, Greece

## Abstract

Background: Tobacco-smoke, airborne, and dietary exposures to polycyclic aromatic hydrocarbons (PAHs) have been associated with reduced prenatal growth. Evidence from biomarker-based studies of low-exposed populations is limited. Bulky DNA adducts in cord blood reflect the prenatal effective dose to several genotoxic agents including PAHs.

Objectives: We estimated the association between bulky DNA adduct levels and birth weight in a multicenter study and examined modification of this association by maternal intake of fruits and vegetables during pregnancy.

Methods: Pregnant women from Denmark, England, Greece, Norway, and Spain were recruited in 2006–2010. Adduct levels were measured by the ^32^P-postlabeling technique in white blood cells from 229 mothers and 612 newborns. Maternal diet was examined through questionnaires.

Results: Adduct levels in maternal and cord blood samples were similar and positively correlated (median, 12.1 vs. 11.4 adducts in 10^8^ nucleotides; Spearman rank correlation coefficient = 0.66, *p* < 0.001). Cord blood adduct levels were negatively associated with birth weight, with an estimated difference in mean birth weight of –129 g (95% CI: –233, –25 g) for infants in the highest versus lowest tertile of adducts. The negative association with birth weight was limited to births in Norway, Denmark, and England, the countries with the lowest adduct levels, and was more pronounced in births to mothers with low intake of fruits and vegetables (–248 g; 95% CI: –405, –92 g) compared with those with high intake (–58 g; 95% CI: –206, 90 g)

Conclusions: Maternal exposure to genotoxic agents that induce the formation of bulky DNA adducts may affect intrauterine growth. Maternal fruit and vegetable consumption may be protective.

Citation: Pedersen M, Schoket B, Godschalk RW, Wright J, von Stedingk H, Törnqvist M, Sunyer J, Nielsen JK, Merlo DF, Mendez MA, Meltzer HM, Lukács V, Landström A, Kyrtopoulos SA, Kovács K, Knudsen LE, Haugen M, Hardie LJ, Gützkow KB, Fleming S, Fthenou E, Farmer PB, Espinosa A, Chatzi L, Brunborg G, Brady NJ, Botsivali M, Arab K, Anna L, Alexander J, Agramunt S, Kleinjans JC, Segerbäck D, Kogevinas M. 2013. Bulky DNA adducts in cord blood, maternal fruit-and-vegetable consumption, and birth weight in a European mother–child study (NewGeneris). Environ Health Perspect 121:1200–1206; http://dx.doi.org/10.1289/ehp.1206333

## Introduction

Environmental exposures *in utero* may have adverse effects on health both immediately and in later life. Measurement of biomarkers in cord blood improves exposure assessment and may improve our understanding of biological mechanisms during this critical window of exposure and vulnerability (Wild and Kleinjans 2003).

Bulky DNA adducts are a widely accepted and sensitive biomarker of the biologically effective dose of genotoxic agents in complex environmental exposures, including those in ambient air, tobacco smoke, and diet (Godschalk et al. 2005; Karttunen et al. 2010; Kovács et al. 2011). They reflect individual exposure, absorption, and metabolic activation of heterogeneous adduct-forming compounds, in combination with the ability to repair induced DNA damage (Farmer 1994), and may be predictive of cancer risk (Veglia et al. 2008).

Bulky DNA adducts are commonly detected in human DNA by ^32^P-postlabeling combined with multidimensional thin-layer chromatography. Among common environmental genotoxic agents, polycyclic aromatic hydrocarbons (PAHs) [International Agency for Research on Cancer (IARC) 2010] cause DNA damage that is readily detectable as bulky DNA adducts, although the chemical nature of the DNA damage that leads to adduct formation is not known with certainty. A positive correlation between DNA adducts in blood and PAH exposure has been reported in adult populations exposed to high levels of PAHs in ambient air or food (Nielsen et al. 1996; van Maanen et al. 1994), which suggests that bulky DNA adducts reflect DNA damage caused by genotoxic PAHs. Bulky DNA adducts and more specific PAH-related DNA adducts have been detected in human umbilical cord white blood cells (Godschalk et al. 2005; Hansen et al. 1993; Pedersen et al. 2009; Perera et al. 1998, 2005; Topinka et al. 2009), in human placenta (Everson et al. 1988; Hansen et al. 1993; Sram et al. 2006) and in *ex vivo* human placental perfusions (Karttunen et al. 2010), which suggests that PAHs and other environmental genotoxic agents are capable of forming DNA adducts *in utero*.

Food is an important source of PAHs (IARC 2010; Kazerouni et al. 2001). Intake of meat with a blackened surface (Pedersen et al. 2012a), exposure to traffic-related air pollution during pregnancy (Pedersen et al. 2009), and smoking during pregnancy (Godschalk et al. 2005; Hansen et al. 1993; Pedersen et al. 2009) have been associated with higher levels of bulky DNA adducts in human cord blood. However, evidence regarding associations between bulky DNA adducts and birth outcomes is conflicting. Smoking-related DNA adducts measured by the ^32^P-postlabeling method in placental tissue from 30 women in the United States were associated with reduced birth weight (Everson et al. 1988), but in a study of 199 women in the Czech Republic, there were no associations between bulky DNA adducts in placenta tissue and birth weight, the risk of low birth weight (< 2,500 g), gestational duration, or preterm delivery (Sram et al. 2006).

Consumption of fruits and vegetables is considered beneficial for health (Slavin and Lloyd 2012) and may protect against cancer (World Cancer Research Fund/American Institute for Cancer Research 2007) through antioxidative effects and other properties related to dietary intake of fiber, folate, and other beneficial nutrients. In the European Prospective Investigation into Cancer and Nutrition (EPIC) cohort study, higher intake of fibers was negatively associated with bulky DNA adduct levels in white blood cells from 1,085 adults (Peluso et al. 2008). In addition, a diet rich in vitamin C has been associated with lower levels of DNA damage [reviewed by Sram et al. (2012)], and high maternal vitamin C intake during pregnancy appeared to reduce the association between estimated maternal dietary benzo[*a*]pyrene (BaP) intake and size at birth of 586 newborns from Spain (Duarte-Salles et al. 2012).

In the present study, we investigated the association between bulky DNA adduct levels and birth weight in 612 newborns and further assessed whether maternal consumption of fruits and vegetables during pregnancy modified this association.

## Methods

*Study population*. The study was conducted as a part of the NewGeneris (Newborns and Genotoxic exposure risks) study of the impact of diet during pregnancy on child health (Merlo et al. 2009). Pregnant women were enrolled during 2006–2010 from 11 maternity units located in Copenhagen, Denmark; Bradford, England; Heraklion, Greece; Oslo, Norway; and Barcelona and Sabadell, Spain (Pedersen et al. 2012b). Births were included in the present analysis if they occurred during the periods of cord blood collection and processing, if there was a sufficient volume of cord blood, and if blood processing and biomarker analysis was successful. Precise participation rates for the present analysis cannot be estimated because the number of births that might have been eligible cannot be determined.

Detailed information on personal characteristics was obtained from the mothers by using extensive questionnaires collected before or around the time of delivery (Table 1). The questionnaires were self-administered (Denmark-2009, Norway), partly supported (Denmark-2007, Spain, and England), and administered by an interviewer (Greece) (Pedersen et al. 2012b). Dietary information concerning diet during pregnancy was obtained from country-specific food frequency questionnaires. Information on birth weight, birth head circumference, gestational age, infant sex, and mode of delivery was obtained from maternity records. Gestational age at birth was based on last menstrual period and/or ultrasound-based estimated date of conception and corrected by ultrasound measurements if there was a discordance of ≥ 7 days between both estimates.

**Table 1:** Study population characteristics.

Characteristic	All (*n* = 612)^*a*^	North (*n* = 367)^*b*^	South (*n* = 245)^*b*^	*p*-Value^*c*^
Country				< 0.001
Greece	68 (11.1)	0 (0.0)	68 (27.8)
Spain	177 (28.9)	0 (0.0)	177 (72.2)
Norway	58 (9.5)	58 (15.8)	0 (0.0)
England	109 (17.8)	109 (29.7)	0 (0.0)
Denmark	200 (32.7)	200 (54.5)	0 (0.0)
Maternal age (years)	32 (15–46)	32 (17–46)	31 (15–46)	< 0.001
White European mother	473 (77.5)	319 (86.9)	154 (63.4)	< 0.001
Maternal education^*d*^				< 0.001
Low	113 (21.9)	59 (18.4)	54 (27.8)
Middle	189 (36.7)	99 (30.8)	90 (46.4)
High	213 (41.4)	163 (50.8)	50 (25.8)
Multiparous mother	390 (65.2)	240 (67.2)	150 (62.2)	0.21
Maternal BMI (kg/m^2^)	22.8 (15.8–56.0)	22.7 (15.8–54.6)	23.1 (16.8–56.0)	0.24
Energy intake (kcal/day)	2,457 (622–5,918)	2,480 (622–5,918)	2,402 (874–5,844)	0.79
Fruits and vegetables (g/day)	579 (0–5023)	557 (0–5023)	615 (0–3387)	0.13
Fruits and vegetables (g/1,000 kcal/day)	235 (0–1099)	221 (0–1065)	260 (0–1099)	0.02
Vitamin C fruit (g/day)	121 (0–1810)	120 (0–1122)	122 (0–1810)	0.26
Vitamin C fruit (g/1,000 kcal/day)	50 (0–490)	48 (0–448)	52 (0–490)	0.35
Dietary supplement intake	436 (87)	271 (85)	165 (90)	0.18
Maternal active smoking^*e*^	71 (11.6)	28 (7.6)	43 (17.6)	< 0.001
Secondhand smoke^*f*^	213 (37.0)	97 (27.5)	116 (52.0)	< 0.001
Ethylene oxide (pmol/g Hb)^*g*^	9.7 (0.5–120.7)	9.9 (0.5–120.7)	9.6 (2.6–88.1)	0.999
Season of delivery				< 0.001
March–May	162 (26.5)	116 (31.6)	46 (18.8)
June–August	90 (14.7)	50 (13.6)	40 (16.3)
September–November	227 (37.1)	151 (41.1)	76 (31.0)
December–February	133 (21.7)	50 (13.6)	83 (33.9)
Vaginal mode of delivery	342 (56.0)	161 (43.9)	181 (74.2)	< 0.001
Male infant	322 (52.6)	187 (51.0)	135 (55.1)	0.31
Gestational age (weeks)	39 (33–43)	39 (35–42)	39 (33–43)	< 0.001
< 37 weeks	26 (4.3)	5 (1.4)	21 (8.6)	
Birth weight (g)	3,440 (2,060–4,700)	3,544 (2,060–4,700)	3,325 (2,190–4,510)	< 0.001
< 2,500 g	7 (1.1)	3 (0.8)	4 (1.6)	< 0.001
Birth head circumference (cm)	35 (30–39)	35 (31–39)	35 (30–38)	< 0.001
Adducts (*n*/10^8^ nucleotides)^*g*^	8.4 (0.6–87.5)	7.0 (0.6–52.7)	12.8 (0.8–87.5)	< 0.001
Values are *n *(%) or median (minimum–maximum). ^***a***^Total in specific variables may be < 612 because of missing values. ^***b***^North refers to Denmark, England, and Norway; South refers to Greece and Spain. ^***c***^*p*-Value from chi-square or Kruskall–Wallis test for North–South comparisons. ^***d***^Country-­specific definition. ^***e***^Women who smoked at end of pregnancy. ^***f***^At home and elsewhere. ^***g***^Measured in cord blood.				

Cord blood DNA adduct measurements were available from 630 newborns born to women with singleton deliveries. We excluded 18 newborns with missing information on maternal smoking, gestational age, birth weight, and/or sex, and included 612 newborns.

Ethical approval was obtained from the ethics committee in each country. Written informed consent was obtained from all participating women.

*Blood collection and bulky DNA adduct analysis (^32^P-postlabeling)*. Umbilical cord blood (~ 50 mL) was collected immediately after delivery. Peripheral blood (~ 45 mL) was also drawn from 229 mothers.

DNA was isolated centrally from ~ 0.5-mL aliquots of buffy-coat samples using Qiagen Midi Kit catalog no.13343 (Qiagen, Hilden, Germany) with some modifications (Arab et al. 2009). Levels of bulky DNA adducts were determined by using the ^32^P-postlabeling method with the nuclease P1 adduct enrichment version according to standardized protocols (Godschalk et al. 2005; Karttunen et al. 2010; Kovács et al. 2011). The protocols were harmonized and adjusted in an interlaboratory comparison study among the three ^32^P-postlabeling investigator laboratories, including the use of the same external benzo[*a*]pyrene-7,8-diol-9,10-epoxide (BPDE)–DNA standard [111 adducts in 10^8^ normal nucleotides (nt), which was a kind gift from F.F. Beland (National Center for Toxicological Research, Little Rock, AR, USA)]. All samples from Greece, Spain, and Norway and the Danish samples collected in 2006–2007 were analyzed in Budapest, Hungary (61% of the samples); the Danish samples from 2009 were analyzed in Stockholm, Sweden (21%); and the samples from England were analyzed in Maastricht, the Netherlands (18%). The interlaboratory comparison study showed a very high repeatability between two of the three laboratories, whereas the adduct levels measured in the third laboratory were consistently 3.7 times lower than the mean levels determined by the two other laboratories. Differences in DNA adduct determinations between laboratories normally occur because of the complicated multistep and sensitive procedures used for the detection of the adducts, and interlaboratory studies are therefore necessary. A correction for the laboratory factor of 3.7 was thus applied to the samples analyzed in Stockholm (Denmark-2009, 21% of the total). A sensitivity analysis that included or subsequently excluded these samples gave similar results, so all analyses were based on the total study population including the corrected data.

The individual level of DNA adducts was obtained as the average of at least two independent measurements. The detection limit of the assay was approximately 0.1–0.3 adducts per 10^8^ unmodified nucleotides (*n*/10^8^ nt).

*Statistical analysis.* We performed linear regression models with a random effect for country to estimate the difference in mean birth weight (grams) associated with bulky DNA adduct levels in cord blood. Furthermore, we estimated associations with head circumference (centimeters) and gestational age (completed weeks) at birth. Low birth weight (< 2,500 g, *n* = 7) and preterm (< 37 completed weeks of gestation, *n* = 26) were too uncommon to estimate associations with bulky DNA adduct levels.

DNA adduct levels measured in cord blood were modeled as categorized according to tertiles: low (*n* = 205), middle (*n* = 203), or high (*n* = 204) (< 5.9, ≥ 5.9–12.4, and ≥ 12.5 adducts/10^8^ nt, respectively).

We examined the effect of different degrees of adjustment for potential confounders on the association of bulky DNA adduct levels on birth weight. Potential confounders selected *a priori* for the adjusted model were gestational age (completed weeks, continuous), infant sex, maternal prepregnancy body mass index (BMI; kilograms per meter squared), parity (0, ≥ 1), maternal age (years), maternal ethnicity (white, nonwhite), self-reported maternal active smoking at the end of pregnancy (no, yes), self-reported maternal exposure to secondhand smoke (SHS) during pregnancy (no, yes), ethylene oxide–hemoglobin (Hb) adduct levels in cord blood (picomoles per gram Hb; to assess exposure to tobacco smoke during pregnancy), mode of delivery (vaginal, cesarean section), dietary supplements (none, any), maternal consumption of fruits and vegetables during pregnancy (low, high), season of delivery (March–May, June–August, September–November, December–February), country of delivery, and maternal education (low, middle, high) as a marker of socioeconomic position. In addition, we estimated basic adjusted associations using models that included country, maternal smoking at the end of pregnancy, sex, and gestational age only.

Given that bulky DNA adduct levels in cord blood reflect a steady state between DNA damage and repair during the previous few months (Godschalk et al. 2005), we classified women who smoked during the last four months of pregnancy as smokers, whereas those who never smoked or who quit before the last 4 months of pregnancy were classified as nonsmokers. Women exposed to SHS in the home and/or elsewhere during pregnancy were categorized as exposed. In addition, to further assess exposure to dietary acrylamide and tobacco smoke during pregnancy, we adjusted for acrylamide and ethylene oxide Hb adduct levels (picomoles per gram Hb), respectively, in cord blood samples (von Stedingk et al. 2011).

Maternal intake of fruits and vegetables during pregnancy (grams/day, based on 20–61 questionnaire items depending on country) was categorized as high or low according to overall and country-specific median levels as a proxy measure of the consumption of nutrients that might be protective against genotoxic activation processes. We also classified mothers according to their intake of fruits high in vitamin C and other antioxidants (i.e., all types of citrus fruits, both in terms of whole fruits and juice, kiwi fruit, and berries, ranging from three to seven questionnaire items). Women (*n* = 54) with a total energy estimate of < 500 or > 6,000 kcal/day were excluded from adjusted analyses and analyses of effect modification by diet (Butte and King 2005; Willett 1998).

In addition, we performed a meta-analysis by country to derive country-specific effect estimates of associations between cord blood DNA adduct levels and birth weight. Pregnancy outcomes in Northern European countries (England, Denmark, and Norway, *n* = 367), which had low average levels of adducts, were compared with outcomes in Southern European countries (Greece and Spain, *n* = 245), which had higher average adduct levels.

To estimate the association between bulky DNA adduct levels and term birth weight, we repeated the main analysis after excluding preterm deliveries (*n* = 26). We used an alpha level of 5% for statistical significance. All statistical analyses were performed using Stata S.E. version 12.1 (StataCorp, College Station, TX, USA).

## Results

*Study population.* The study population was composed of neonates from Denmark (33%), Spain (29%), England (18%), Greece (11%), and Norway (10%). Mothers were predominantly white Europeans, multiparous, and nonsmoking (Table 1). Most children were born at term (96%) and weighed > 2,500 g at birth (97%). Some study population characteristics differed significantly between the Northern and Southern European populations (e.g., maternal smoking; 8% vs. 18%, respectively), whereas characteristics such as maternal prepregnancy BMI and dietary supplement use were similar. The daily median fruit and vegetable intake of the Southern European mothers was 58 g higher than that of Northern European mothers, but the difference was not statically significant (*p* = 0.13). The difference in fruit and vegetable intake between Southern and Northern European mothers was also not statically significant after adjustment for individual total energy intake. Differences were smaller for fruits high in vitamin C (Table 1).

*Adduct levels in maternal and cord blood samples.* All maternal (*n* = 229) and cord blood (*n* = 612) samples had detectable levels of adducts. Median levels of adducts in paired maternal and cord blood samples were similar (12.1 vs. 11.4 adducts/10^8^ nt, *p* = 0.23). Cord blood adduct levels ranged from 0.6 to 87.5 (adducts/10^8^ nt) and were significantly positively correlated with maternal levels (Spearman’s rank correlation coefficient = 0.66, *p* = 0.001, *n* = 229).

**Figure 1:**
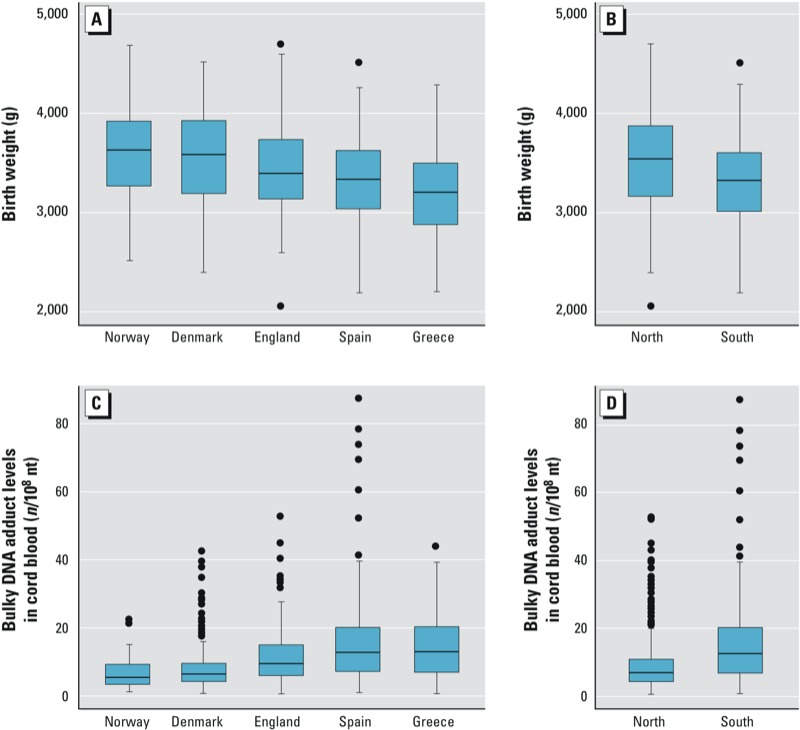
Birth weight (*A–B*) and bulky DNA adduct levels in cord blood (*C–D*):­distribution by country (*A,C*) and region (*B,D*). The horizontal line is the median, and the bottom and top of the box are the first and third quartiles. Whiskers indicate the variability outside the upper and lower quartiles (i.e., within 1.5 interquartile range of the lower quartile and upper quartile). Outliers are plotted as individual dots.

Bulky DNA adduct levels were higher in children from Southern Europe (median, 12.8 adducts/10^8^ nt; range, 0.8–87.5) than from Northern Europe (median, 7.0/10^8^ nt; range, 0.6–52.7; *p* < 0.001), although an opposite pattern was observed for birth weight (medians of 3,325 g and 3,544 g, respectively, *p* < 0.001) (Table 1, Figure 1). The difference in DNA adduct levels was also observed when the analysis was restricted to children born to mothers who did not smoke during the last 4 months of pregnancy (median, 13.0/10^8^ nt; range, 0.8–87.5 for Southern Europe vs. median, 6.8/10^8^ nt; range, 0.6–52.7 for Northern Europe; *p* < 0.001). Median adduct levels in the children of nonsmokers also differed significantly (*p* < 0.001) among the individual countries (Greece: 13.4/10^8^ nt, range 0.8–43.9, *n* = 54; Spain: 12.9/10^8^ nt, range 1.1–87.5, *n* = 148; England: 9.5/10^8^ nt, range 0.6–52.7, *n* = 57; Denmark: 6.4/10^8^ nt, range 1.3–42.7, *n* = 192; Norway: 5.4/10^8^ nt, range 1.2–22.3, *n* = 57). The same pattern of higher adduct levels in children from Southern Europe than those from Northern Europe was found for children born to nonsmokers without exposure to SHS.

The median bulky DNA adduct level in the 71 children born to mothers who actively smoked at the end of their pregnancy (10.9 adducts/10^8^ nt; range, 0.6–73.9) was higher than the median level in the 541 children of mothers who never smoked or who quit before the last 4 months of pregnancy (8.2 adducts/10^8^ nt; range, 0.6–87.5, *p* = 0.07). Adduct levels were lowest in the 505 children born to mothers who never smoked during their pregnancy (8.0 adducts/10^8^ nt; range, 0.6–87.5, *p* = 0.10).

*Adduct levels in cord blood and birth outcomes.* Higher levels of bulky DNA adducts in cord blood were associated with lower birth weight (Table 2). For the full study population (*n* = 612), the estimated difference in mean birth weight for infants in the highest tertile versus the lowest tertile of adduct levels was –110 g (95% CI: –192, –28 g), based on basic adjusted models that included country, maternal smoking at the end of pregnancy, sex, and gestational age only. The corresponding association was similar when restricted to the 541 mothers who did not smoke during the last 4 months of pregnancy (–108 g; 95% CI: –202, –14 g), but was slightly stronger when restricted to the 505 mothers who did not smoke at any time during the pregnancy (–124 g; 95% CI: –216, –32 g).

**Table 2:** Change in birth weight (g) associated with in cord blood bulky DNA adduct levels.

Variable	*n*	β (95% CI)	*p*-Value
Basic adjusted^*a*^
Adducts (increase of 10 adducts/10^8^ nt)	612	–30 (–62, 2)	0.07
Low (< 5.9/10^8^ nt)^*b*^	205	Reference
Middle (5.9–12.4/10^8^ nt)	203	–47 (–128, 35)	0.26
High (> 12.4/10^8^ nt)	204	–110 (–192, –28)	0.009
Further adjusted^*c*^
Adducts (increase of 10 adducts/10^8^ nt)	409	–21 (–62, 21)	0.32
Low (< 5.9/10^8^ nt)^*d*^	153	Reference
Middle (5.9–12.4/10^8^ nt)	140	–51 (–146, 43)	0.29
High (> 12.4/10^8^ nt)	116	–129 (–233, –25)	0.015
^***a***^Effect estimates on birth weight (g) in linear regression models adjusted for gestational age, infant sex, maternal active smoking at the end of pregnancy, and country (random effect). ^***b***^The mean birth weight of the reference group was 3,510 g. ^***c***^Further adjusted for maternal ethnicity, maternal pre­pregnancy BMI, parity, maternal age, maternal exposure to SHS, mode of delivery, maternal education, maternal consumption of fruits and vegetables, and season of delivery. ^***d***^The mean birth weight of the reference group was 3,559 g.			

After further adjusting for maternal age, prepregnancy BMI, exposure to SHS, maternal education, ethnicity, intake of fruits and vegetables, delivery type, and season of delivery (*n* = 409), the estimated difference in mean birth weight associated with the highest versus lowest tertile of DNA adduct levels was –129 g (95% CI: –233, –25 g) (Table 2). After additional adjustment for ethylene oxide–cord blood Hb adduct levels (picomoles per gram Hb, *n* = 390), the estimated mean difference was –139 g (95% CI: –245, –32 g). When we further adjusted for acrylamide Hb adduct levels in cord blood (picomoles per gram Hb, *n* = 390) the associations remained practically unchanged (–140 g (95% CI: –247, –34 g).

When 26 preterm births (< 37 weeks) were excluded, the estimated difference in birth weight for infants in the highest versus lowest tertile of adduct levels was –139 g (95% CI: –245, –33 g).

Bulky DNA adduct levels were negatively associated with head circumference based on the basic adjusted model (–0.28 cm for the highest vs. lowest tertile; 95% CI: –0.59, 0.03 cm, *p* = 0.08, *n* = 530) and after further adjustment for maternal age, prepregnancy BMI, exposure to SHS, maternal education, ethnicity, intake of fruits and vegetables, delivery type, and season of delivery (–0.33 cm; 95% CI: –0.72, 0.06 cm, *p* = 0.10, *n* = 388).

The estimated difference in mean gestational age at birth for infants in the highest versus lowest tertile of adduct levels was –0.29 weeks (95% CI, –0.63, 0.04 weeks; *p* = 0.08) based on the further adjusted model. When restricted to vaginal deliveries (*n* = 243, further adjusted model), the estimated difference in gestational age was –0.54 weeks (95% CI: –1.06, –0.03 weeks), compared with a difference of 0.15 weeks (95% CI: –0.23, 0.53 weeks; *p* = 0.43) for deliveries by cesarean section (*n* = 166).

**Figure 2:**
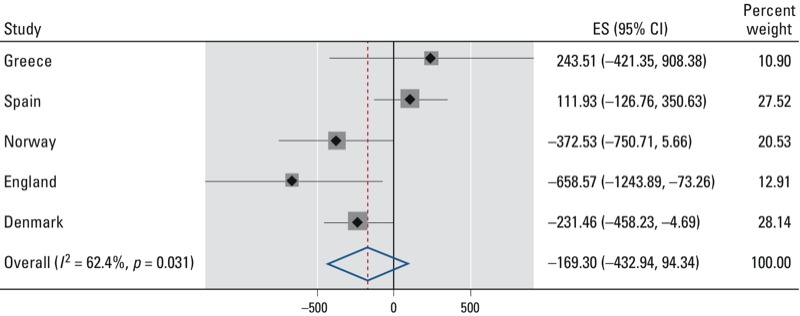
Change in birth weight (g) associated with the bulky DNA adduct levels in cord blood (per 10^8^ nt) by country. Country-specific effect estimates (ES) and their 95% CIs as well as the meta-analyses combined effect estimates (random effect of country), which correspond to the change in birth weight for the highest relative to the lowest tertile of cord blood bulky DNA adduct levels further adjusted as described in Table 2 (*n* = 409). Black diamonds indicate ESs; gray squares are proportional to the country-specific weights used in the meta-analyses, and the associated 95% CIs are shown as horizontal black lines. The summary ES, which corresponds to the change in birth weight (g), is indicated with a red dashed vertical line and blue diamond, and the associated 95% CIs are indicated by the lateral tips of the diamond. The solid vertical black line indicates no change in birth weight.

*Differences between countries.* Consistent with associations estimated for the full study population, mean birth weight was significantly lower for Northern European infants in the highest versus lowest tertile of adduct levels (–119 g; 95% CI: –234, –4 g) based on the basic adjusted model. However, there was a nonsignificant positive association among the 245 Southern Europeans (71 g; 95% CI: –59, 202 g; *p* = 0.28). Estimates based on adjusted models also indicated negative associations for the Northern European countries, but not for the Southern European countries (Figure 2; *p*-value for heterogeneity = 0.03), and when imputed data were used to include individuals with missing information on covariates (results not shown). Therefore, although birth weight was lowest and adduct levels were highest in Greece and Spain (Figure 1), the negative association between adduct levels and birth weight estimated for the full study population appears to be driven by the Northern countries.

*Differences by intake of fruits and vegetables.* The association of bulky DNA adduct levels with birth weight differed according to maternal intake of fruits and vegetables and intake of fruits high in vitamin C, although interactions were only marginally significant (Table 3). The estimated difference in birth weight between the highest and lowest tertiles of adduct levels was greater among births to mothers with low intakes of fruits and vegetables (–248 g; 95% CI: –405, –92 g) than among births to mothers with high intakes (–58 g; 95% CI: –206, 90 g; *p* = 0.44). Consumption of dietary supplements during pregnancy was common in both Northern and Southern Europeans (85% and 90%, respectively) and did not appear to modify or confound associations between adduct levels and birth weight (data not shown).

**Table 3:** Modification of the change in birth weight (g) associated with bulky DNA adduct levels by maternal intake of fruits and vegetables during pregnancy.

Models	Low maternal intake^*a*^			High maternal intake			*p-*Value^*c*^
	*n*	β^*b*^ (95% CI)	*p-*Value	*n*	β (95% CI)	*p-*Value	
Fruits and vegetables
Adducts (increase of 10 adducts/10^8^ nt)	197	–22 (–80, 36)	0.45	212	–22 (–86, 42)	0.51	0.77
Low (< 5.9/10^8^ nt)^*d*^	71	Reference		82	Reference		
Middle (5.9–12.4/10^8^ nt)	71	–78 (–217, 61)	0.27	69	–37 (–173, 100)	0.60	0.75
High (> 12.4/10^8^ nt)	55	–248 (–405, –92)	0.002	61	–58 (–206, 90)	0.44	0.077
Fruit high in vitamin C
Adducts (increase of 10 adducts/10^8^ nt)	201	–39 (–94, 15)	0.15	208	3 (–64, 70)	0.93	0.63
Low (< 5.9/10^8^ nt)^*e*^	66	Reference		87	Reference		
Middle (5.9–12.4/10^8^ nt)	73	–120 (–259, 19)	0.09	67	–0 (–129, 128)	1.00	0.54
High (> 12.4/10^8^ nt)	62	–266 (–421, –112)	0.001	54	–39 (–186, 107)	0.60	0.26
^***a***^Low corresponds to < 579 g/day, which is the overall median intake; high corresponds to ≥ 579 g/day in terms of fruits and vegetables. For fruit high in vitamin C, low corresponds to < 121 g/day and high corresponds to ≥ 121 g/day. ^***b***^Effect estimates on birth weight (g) in linear regression models further adjusted; see Table 2. ^***c***^*p*-Value for the interaction term between maternal intake (low, high) and bulky DNA adduct level in cord blood. ^***d***^The mean birth weight of the reference group was 3,613 g for low intake and 3,513 g for high intake. ^***e***^The mean birth weight of the reference group was 3,593 g for low intake and 3,534 g for high intake.							

## Discussion

We measured levels of bulky DNA adducts in white blood cells from cord blood in a large multicenter European prospective general population study and found that higher adduct levels in cord blood were significantly negatively associated with birth weight. The negative association was observed among newborns from England, Denmark, and Norway, who had the lowest average adduct levels, but was not evident among Southern Europeans, who had the highest mean adduct levels. The negative association with birth weight was stronger among the children of mothers with low versus high intakes of fruits and vegetables.

Tobacco smoke contains PAHs and other DNA adduct–forming compounds in addition to other potentially harmful compounds, and active maternal smoking is a recognized risk factor for reduced fetal growth (Li et al. 1993). Furthermore, ambient airborne PAHs (Wilhelm et al. 2011) and intake of barbecued meat during pregnancy has also been associated with reduced birth weight (Jedrychowski et al. 2012).

The results of the previous similar biomarker-based studies on fetal growth are inconsistent; studies based on 1-hydroxypyrene in maternal urine (*n* = 449 samples, Poland) (Polańska et al. 2010), bulky DNA adducts (*n* = 30, newborns of smokers, United States) (Everson et al. 1988), and structurally related PAH–DNA adducts in cord blood (*n* = 135, newborns of women living in coal-burning areas, Poland) (Perera et al. 1998) support an association between higher prenatal exposure to PAHs and reduced birth weight opposite to the findings from cord blood–based studies on bulky DNA adducts (Sram et al. 2006), BPDE–DNA adducts (*n* = 181, newborns of women who lived near the World Trade Center fires on 11 September 2001 while they were pregnant) (Perera et al. 2005) and on BPDE–DNA adducts (*n* = 150, newborns of women living near a coal-fired power plant, China) (Tang et al. 2006). Results from these studies are, however, inconsistent, and evidence from European populations exposed to contemporary lifestyle and environment is limited.

Mechanisms by which environmental genotoxicants that cause bulky DNA adduct formation might affect fetal growth are not known; but along with direct modification of DNA (measurable as bulky DNA adducts), possible mechanisms may include binding to aryl hydrocarbon receptor (AhR) and/or other receptors causing endocrine disruption, altered placental growth, decreased placental exchange of nutrients and gases *in utero* possibly related to induction of P450 enzymes, global DNA methylation changes, induction of apoptosis, altered gene expression, cellular mutations, or oxidative stress (Baird et al. 2005; IARC 2010; Kannan et al. 2006; Sram et al. 2006; Tang et al. 2012). Developmental and reproductive toxicity due to prenatal exposure to PAHs and similar AhR ligands has been observed in various animal species (Pocar et al. 2005; Sanyal and Li 2007).

Our findings suggest that maternal intake of fruits and vegetables may modify the association between bulky DNA adduct levels and birth weight. This finding is consistent with the findings of a questionnaire-based study evaluating dietary BaP (Duarte-Salles et al. 2012).

Children from Southern Europe had, on average, higher bulky DNA adduct levels and lower birth weight than Northern European children. However a negative association between adducts and birth weight was found only in the Northern Europeans. The same pattern of higher bulky DNA adduct levels in Southern Europe than in Northern Europe has been found in adults (Ricceri et al. 2010), and this may reflect differences in ambient air quality (Table 4), but also wider geographical differences in diet, food preparations, and other factors, or perhaps different susceptibility toward environmental genotoxic agents. It is possible that exposures that cause adducts in children from Northern Europe also cause reduced birth weight, whereas exposures responsible for adducts among Southern European children might differ and may not affect birth weight. A complementary explanation could involve a saturation effect of the toxicity of these adducts on birth weight, although other studies in populations exposed to very high air pollution levels and corresponding high adduct levels have reported negative associations with birth weight (Perera et al. 1998, 2005).

**Table 4:** Annual mean of ambient air pollution (μg/m^3^).

Location	Year	NO_2_	PM_10_	PM_2.5_	Reference
Denmark, Copenhagen	2007	19	24	10	DCE 2013
Denmark, Copenhagen	2009	18	21	11	DCE 2013
Norway, Oslo and Akershus	2008	38	11	10	NILU 2013
England, Bradford	2008	25	NA	NA	CBMDC 2009
Spain, Sabadell	2007	29	40	18	Gencat 2013a, 2013c, 2013e
Spain, Barcelona	2009	40	34	20	Gencat 2013b, 2013d, 2013f
Greece, Heraklion	2007	41	20	NA
Abbreviations: CBMDC, City of Bradford Metropolitan District Council; DCE, Danish Center for Environment and Energy at Aarhus University; Gencat, Generalitat de Catalunya; NA, not available; NILU, Norsk institutt for luftforskning; NO_2_, nitrogen dioxide; PM_10_, particulate matter with an aerodynamic diameter ≤ 10 μm; PM_2.5_, particulate matter with an aerodynamic diameter ≤ 2.5 μm.					

A key strength of the present study is the measurement of bulky DNA adducts in cord blood, which enabled a more accurate evaluation of the biologically effective dose of genotoxic agents resulting from complex environmental exposures than estimates based solely on external exposure assessment. Large biomarker-based studies are rare because of their costs and complexity. Standardized protocols were developed (Merlo et al. 2009) and applied to the collection of cord blood from multiple study centers. Detailed information on maternal characteristics and diet was collected in a manner that allowed pooling of data from five different countries (Pedersen et al. 2012b). In addition to increasing the sample size, enrolling participants from different countries allows us to test hypotheses in different settings.

^32^P-Postlabeling is considered to be the most suitable and sensitive method for assessing total DNA damage resulting from exposures to unknown, complex mixtures of genotoxic compounds. We used the validated *in vitro* BPDE–DNA standard in each ^32^P-postlabeling session in duplicate for normalization of the DNA adduct levels. Each DNA sample was analyzed at least twice. The interlaboratory comparison study, the use of common protocols, and use of a validated BPDE–DNA standard minimized the potential for measurement error. Thus, we believe that measurement error of the method was properly handled and is not a special concern in our study.

Low birth weight is an important outcome because it is associated with greater risk of neonatal mortality, hypertension and cardiovascular disease, diabetes, certain cancers, reduced and/or delayed postnatal growth, and cognitive development (Gluckman et al. 2008). Our findings suggest that environmental exposures that result in the *in utero* formation of bulky DNA adducts also may affect prenatal growth, and that this potential effect may be reduced by high maternal fruit and vegetable consumption.
